# Mental Health Status and Its Impact on TB Treatment and Its Outcomes: A Scoping Literature Review

**DOI:** 10.3389/fpubh.2022.855515

**Published:** 2022-05-31

**Authors:** Charles Kwaku Agbeko, Manthar Ali Mallah, Biyu He, Qiao Liu, Huan Song, Jianming Wang

**Affiliations:** ^1^Department of Epidemiology, Center for Global Health, School of Public Health, Nanjing Medical University, Nanjing, China; ^2^College of Public Health, Zhengzhou University, Zhengzhou, China; ^3^Key Laboratory of Infectious Diseases, School of Public Health, Nanjing Medical University, Nanjing, China

**Keywords:** tuberculosis, mental health, health-related quality of life, treatment, adherence

## Abstract

**Background:**

Tuberculosis (TB) infection interferes with the health-related quality of life (HRQOL), including physical, social, mental, emotional and financial domains of individuals. The goal of this scoping review is to outline the most frequent mental issues encountered by TB patients and evaluate the effects of mental health on TB treatment outcomes. Our findings identify research gaps that could help bridge the overall treatment outcomes in the near future.

**Method:**

A systematic stepwise approach was adopted to search online resource databases like PubMed, Web of science, and gray literature to retrieve published scientific articles for the review. Titles and abstracts of selected studies were examined for their possible eligibility. The studies matching our eligibility criteria were taken into account for this scoping review.

**Results:**

One hundred and ninety three articles were retrieved out of which 26 met the final inclusion criteria. We found that studies adopting interventional approaches reported good mental wellbeing outcomes and better medical compliance as compared with studies that just investigated the subjects. The data represented 15 countries including three low-income countries (LICs), four low-middle-income-countries (LMICs), seven upper-middle-income countries (UMICs), and one high-income country (HIC).

**Conclusion:**

Depression, anxiety, and poor social support, and stigma affect the wellbeing of individuals across the globe irrespective of age, race, demographic characteristics, geographical location, and social status.

## Introduction

Ever since its discovery and first global outbreak in the year 1882 (1), tuberculosis (TB) has been the leading infectious disease cause of death worldwide and remains a public health concern (2). A report released by the World Health Organization (WHO) estimated the TB-related death toll at 1.3 million as of 2017 with a projection of millions more getting infected in subsequent years (3). Despite the global implementation of Directly Observed Therapy, Short Course (DOTS), TB incidence is decreasing by only 1–2% per year (4). This is substantially lower than the estimated speed through mathematical modeling. The World Health Organization (WHO) clearly defines health as an overall state of complete physical, mental and social wellbeing and not merely the absence of any infirmity or disease (5). TB infection interferes with the health-related quality of life (HRQOL), including physical, social, mental, emotional and financial domains of individuals (6). Adherence and HRQOL are both important indicators of the overall TB treatment spectrum. Evaluating psychiatric comorbidities in HRQOL is needed alongside the diagnosis of TB and likewise assessment with the continuity of anti-TB treatment (7).

Major mental health problems associated with TB are anxiety and depression (8). Anxiety disorders are characterized extensively by excessive worrying, apprehensiveness, the fear of future events, and uneasiness (9). Anxiety disorders make up one of the most common complications of psychiatric disorders. In 2013 alone, it was estimated that 8.2 million cases were reported in the general population of the UK with women being twice the number of people becoming anxious than men (10). Depression on the other hand is a common mental disorder that allows people to experience low mood, loss of self-interest or pleasure, the feeling of guilt or low self-worth, disturbed sleep or appetite, low energy, and poor concentration (11). According to the estimation by WHO, 300 million people are currently living with depression (12) and with no doubt, most people falling in this category are likely to be a patient suffering from TB or other chronic diseases (12). This has been reported in many studies that there is a three to six times increase in the tendency of becoming depressed due to TB and associated co-morbidities (13). These co-conditions pose a greater public health threat because it may lead to medication default.

However, psychological issues complicate treatment outcomes in people with infectious diseases like TB and chronic diseases such as diabetes, multiple sclerosis, Alzheimer's, HIV, and cancer (14). Maneuvering the gaps between the physical, mental and social aspects of health will affect the overall outcome of diseases. Therefore, assessing the association of TB on the mental wellbeing of its victims is usually underestimated in most institutions. Moreover, social support and stigma could be an important factor in the treatment outcomes of TB patients, leading to a decrement in mental wellbeing (15). The socioeconomic burden on households and individuals could pose a devastating effect if a strong social network within one's outreach is lost. Social support is defined as various means by which individuals help others or social interactions making the subject or individual believe he/she is being valued and cared for (16). Social support helps individuals to seek the appropriate and timely help when conditions and network interactions are strong and reliable for psychological support through stressful events especially when it's solely demonstrated through health-seeking behaviors (17). Stigma on the other hand is defined as a mark of disgrace or a form of disgrace associated with a particular circumstance. It's a process that usually begins when a trait of an individual is identified as disvalued or undesirable (15). This is a complex setting involving the community, institutions, and intrapersonal relationships leading to decrement of self-worth and self-value for one self (18).

The goal of this scoping review is to outline the most frequent mental issues encountered by TB patients and evaluate the effects of mental health on TB treatment outcomes. Our findings identify research gaps that could help bridge the overall treatment outcomes in the near future.

## Materials and Methods

### Search Strategy and Study Selection

**Figure 1:**
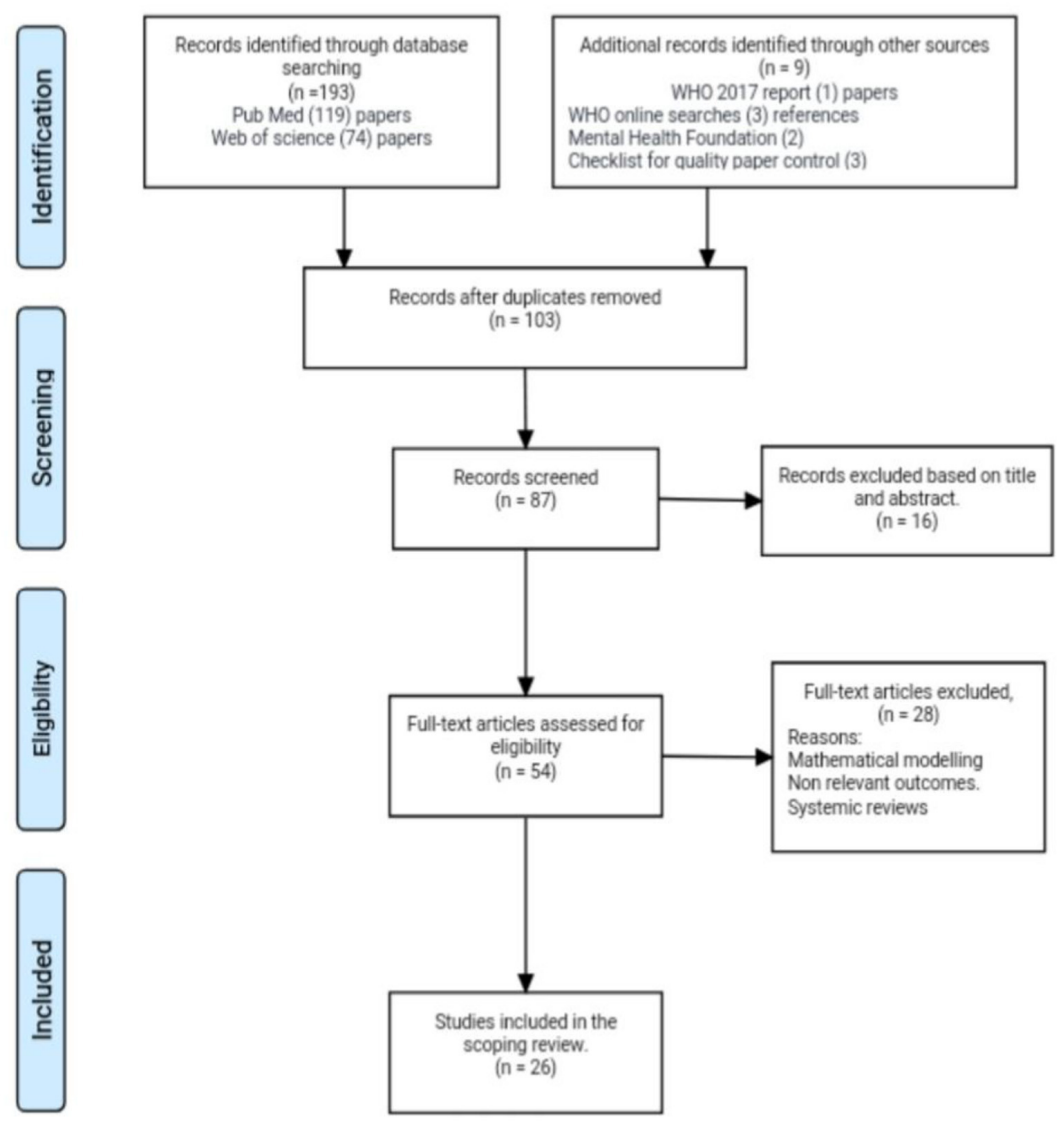
Preferred Reporting Items for Systematic Reviews and Meta Analyses (PRISMA) flow diagram for scoping review.

We systematically searched for studies in PubMed, Web of Science, and the gray literature. A systematic stepwise approach based on the methodological framework proposed by Arksey and O'Malley in 2005 (19) was adopted. An incorporated recommendation by Levac was also used to make the search more comprehensive (20) and up to date. Additionally, the Preferred Reporting Items for Systematic Reviews and Meta-analysis (PRISMA) (21) was employed to guide this review. The search approach was updated to February 2018 and publications in English were included. Keywords for database searching included “mental health status”, “mental health”, “treatment outcomes”, “social support”, “depression”, “anxiety”, “stigma”, “tuberculosis”, and “Mycobacterium tuberculosis” amongst others (full search strategies are detailed in Supplementary File 1). Titles and abstracts of selected studies were examined for their possible eligibility. The studies matching our eligibility criteria were taken into account for this scoping review. Studies were required to have all of the following characteristics: Papers included in our research included both qualitative and quantitative if they fit the mental health status. Articles were also included if they conformed with the search terms of TB and mental health co-morbidity with HIV/AIDS since the majority of studies showed that most people in Africa living with HIV are co-infected with TB. The paper selection flow chart is shown in Figure 1.

### Data Extraction

At each stage of review, two reviewers extracted data independently and they subsequently met to resolve discrepancies. In case of continued disagreement, a third reviewer adjudicated study inclusion. The information extracted from the selected papers included Author(s) name, year of publication, country, aim or purpose of the study, the sample size of population, methodology, intervention type (if applicable), implications, concept, and key findings (Table 1). Supplementary File 2 summarizes the data extracted for this scoping review.

**Table 1:** Data extraction summary.

**#**	**References**	**Year**	**Country**	**Method**	**Size**	**Objective**	**Intervention**	**Findings**	**Implications**
1	Fahad D Alosaimi	2014	Saudi Arabia	Case Report	1	A case of anxiety associated with military tuberculosis	YES	Association of TB causing mental disorders.	When TB was treated, it elevated the metal disorders.
2	Fentie Ambaw	2015	Ethiopia	Prospective cohort design	703	Examine the relationship between depression and TB among people newly diagnosed and accessing care for TB in a rural Ethiopian setting	NO	A sparse evidence based on mental health, TB and other chronic diseases in low-middle income countries	Anticipated impact
3	Muhammad Atif	2014	Malaysia	Prospective follow up	216	Follow up to measure the HRQOL of TB patients	YES	Compromised physical and mental health among study patients even at the end of their TB treatment.	Timely actions for addressing physical and/or mental well-being of the patients.
4	Sushil C Baral	2007	Nepal	Qualitative study	34	To take the first steps toward determining the causes of discrimination associated with TB.	NO	Causes of self-discrimination identified included fear of transmitting TB, and avoiding gossip and potential discrimination.	A comprehensive package of interventions, tailored to the local context, will be needed.
5	K. Chalco	2006	Peru	Qualitative study	Focus groups	To identify the forms and means of emotional support that nurses provide to patients living with multidrug-resistant tuberculosis (MTR-TB)	YES	An emotional support provided by both formal and informal means.	The nurse as a provider of emotional support in the development or implementation of similar programs.
6	M.J. Chinouya	2017	Africans (Zm = 3; Ug =3; Ni =2; Gh=1; Sd=1).	Qualitative study	10	To explore the experiences and meanings of stigma among African men with a previous TB diagnosis.	NO	Men were unable to recognize TB symptoms and subsequently made late clinical presentation when they were also diagnosed with HIV	Multidisciplinary teams supporting ongoing TB education programs should include African men's organizations.
7	Dos Santos	2017	Brazil	Cross sectional	750 beds	To evaluate the HRQL and the prevalence of symptoms of depression and anxiety in hospitalized patients with TB.	NO	Found a possible high prevalence of depression and anxiety in this population.	Health care workers should be aware of these psychological disorders to enable a better management of these patients
8	Juman Abdulelah Dujaili	2015	Iraq	Prospective cohort study	18 years and older	To determine how tuberculosis (TB) treatment affects the health-related quality of life (HRQL) of patients with pulmonary TB and to identify the predictors of favorable TB treatment outcomes in Baghdad, Iraq.	NO	FACIT-TB is a reliable tool to monitor HRQL during the course of TB treatment.	Therapeutic intervention had a positive impact on patient HRQL
9	Bereket Duko	2015	Ethiopia	Cross-sectional study	417	To assess prevalence and correlation of depression and anxiety among patients with TB	NO	The prevalence of depression and anxiety among patients with TB were 43.4 % (181) and 41.5 % (173) respectively.	Developing guidelines and training of health workers in TB clinics is useful
10	Ian F. Walker	2018	Nepal	Mixed quantitative and qualitative approach	135	Assessed the feasibility and acceptability of a psychosocial support package for people receiving treatment for MDR-TB in Nepal.	YES	Psychosocial support package is acceptable to patients	This requires additional investment of counselors in TB clinics.
11	Mohammed O Husain	2008	Pakistan	Cross sectional	108	To determine the presence of depression, anxiety and illness perceptions in patients suffering from Tuberculosis (TB) in Pakistan.	NO	Found that about a half of patients in our sample met the criteria for probable depression and anxiety based on HADS score.	Depression and lack of perceived control are predictors of poor adherence
12	Petros Isaakidis	2013	India	Qualitative study	12	To understand patients' challenges in adhering to treatment for MDR-TB/HIV	YES	Family caregivers were crucial to maintaining the mental and physical health of patients, but reported high levels of fatigue and stress.	Requires high levels of support from family and caregivers to encourage patient adherence and retention in care.
13	Ammar Ali Saleh Jaber	2016	Yemen	Prospective study	243	To evaluate and to find the factors influencing the HRQoL of TB patients in two major TB-prevalent cities.	NO	Highly risk depression was found among TB patients	Efforts should be made to improve their QoL.
14	Tanja Kastien-Hilka	2017	South Africa	Observational longitudinal study.		The aim of this study was to assess the overall impact of TB on the health status	NO	HRQOL of the study participants was impaired in all physical, mental and psycho-social health domains at treatment start but improved during the course of standard TB treatment	The need for an integrative understanding of TB with HRQOL as core element.
15	Jean P. Mukasa	2015	Malawi Australia	Cross sectional study	35 Australian and 104 Malawian	To investigate differences in satisfaction rates among ethnically similar and different patients coming from two dissimilar health systems.	NO	Malawians were mostly inpatients, with recurrent TB episodes, and were more seriously ill with impaired physical and mental wellbeing.	Suggests that patients coming from similar ethnic backgrounds may express similar satisfaction.
16	Olanrewaju Oladimeji	2016	Nigeria	Cross sectional study	98	To assessed the psychosocial wellbeing of multidrug resistant TB (MDR-TB) patients in voluntary and isolated long-term hospitalization in Nigeria.	NO	Prolonged hospitalization resulted insignificant psychosocial burden for the MDR-TB patients in our study centers	There is a need to consider alternative approaches that place less psychosocial burden on patients without compromising quality of care.
17	Benvinda Xavier Paulo	2016	Angola	Cross-sectional study	81	To determine levels of anxiety, depression and emotional distress in patients with several types of TB	NO	Found high rates of anxiety, depression and emotional distress among TB patients.	Mental health services should be an integral part of TB programs.
18	Valerie A Paz-Soldán	2013	Peru	Qualitative	43	To understand psychosocial wellbeing during treatment.	NO	Patients described the need for psychosocial support to mitigate the difficulty of continually going to the clinic to take medications.	Extending educational opportunities to patients' families and the wider community.
19	Karl Peltzer	2012	South Africa	Cross sectional	4,900	Assessed the prevalence and predictors of psychological distress as a proxy for common mental disorders among tuberculosis (TB) patients in South Africa, where over 60 % of the TB patients were co-infected with HIV.	NO	High rates of psychological distress among tuberculosis patients	Improved training of providers in screening for psychological distress.
20	Grant Theron1	2015	Zambia Zimbabwe Tanzania South Africa	Randomized (1:1), parallel-arm, multi-centric trial	-	Correlation of psychological distress and their association with non-adherence to anti-TB treatment.	NO	Severe psychological distress is frequent amongst TB patients in Southern Africa	Targeted interventions to alleviate psychological distress, alcohol use, and improve health literacy in newly-diagnosed TB patients.
21	Cesar Ugarte-Gi	2013	Peru	Longitudinal study	325	To estimate the significance and magnitude of major depressive episode as a hazard factor for negative outcomes.	YES	The presence of MDE at baseline is associated to negative outcome of TB treatment	Targeting detection and treatment of MDE may improve TB treatment outcomes.
22	Annelies Van Rie	2008	Thailand	Cross sectional	47 HIV;480 TB	To develop scales to measure tuberculosis and HIV/AIDS stigma in a developing world context.	YES	Identified two sub-scales associated with both tuberculosis and HIV/AIDS stigma: community and patient perspectives	Their use will help document the burden of stigma.
23	Paulo Benvinda Xavie	2015	Angola	Cross sectional	81	To determine levels of anxiety, depression and emotional distress in patients with several types of TB and to determine the association between social-demographic and economic factors, clinical variables and anxiety, depression and emotional distress.	NO	Found high rates of anxiety, depression and emotional distress among TB patients.	Mental health services should be an integral part of programs against tuberculosis.
24	Shaoru Zhang	2016	China	Qualitative studies	22	To explore the overall illness experience of Chinese high school TB patients and to investigate the individual and social causes of such experience.	NO	Their serious lack of awareness of TB, caused by the ignorance of school, parents and the students, becomes the biggest obstacle to timely diagnosis and treatment	Educational and medical institutions should develop more effective TB Control strategies based on these factors.
25	Shao-Ru Zhan	2010	China	Qualitative studies	17	To explore the experiences and psychological process of college students with pulmonary tuberculosis in Shaanxi, China.	NO	Psychological pressure was significant during the treatment	Colleges should follow governmental policies on TB.
26	Tanja Kastien-Hilka	2017	South Africa	Observational longitudinal	131	Aimed to evaluate the association between HRQOL and adherence in TB patients in South Africa	YES	HRQOL improved over 6-month TB treatment	HRQOL is affected by a number of different factors and not limited to effects of adherence.

### Quality Control

The quality of the articles retrieved was evaluated using different tools. Cross-sectional and observational articles included in this review were cross-checked with the STROBE (strengthening the reporting of observational studies in epidemiology) (22). Longitudinal designs were subjected to a check using the PICO (P stands for population, I stands for intervention, C stands for control and O stands for outcome) (23). Qualitative studies were identified and were also cross-checked by critical appraisal checklist (24). This article was also guided by adopting the PRISMA (21) checklist. The data we retrieved were separated into anxiety, depression and social support, and stigma. The quality check was scored with a reference range of 0–5, where 0 was the least score if articles didn't meet the quality tool checklist and 5 was the highest score if the articles meet the checklist protocol. The three main categorized articles were then combined in one folder using endnote to check for duplications of articles. All studies included in this review met the quality assessment framework of their respective studies. Table 2 shows the quality assessment for eligible studies included in Data extraction.

**Table 2:** Quality assessment for eligible studies included in data extraction.

**Type of studies**	**Number of studies**	**Article number**	**Checklist**	**Reference score (0–5)**
Qualitative studies	7	4, 5, 6, 12, 18, 24, 25	Glasgow Critical Appraisal	4.0
Cross sectional	9	7, 9, 11, 15, 16, 17, 19, 22, 23	STROBE	4
Cohort studies	3	2, 8, 13	STROBE	4.5
Mixed qualitative and quantitative studies	1	10	-	-
Case report	1	1	-	-
Follow up	1	3	-	-
Longitudinal studies	3	14, 21, 26	PICO	4.5
Randomized	1	20	-	-

## Results

### Study Selection

The results primarily yielded an overall number of 1,972 papers during the initial hit in the search engines. We then applied the combination of the mesh terms to yield 193 papers. Our online search also yielded two links from the mental health foundation, three quality checklist kits, three WHO online searches for statistics on depression, MDGs, and SDGs, and one pdf file from the current WHO report on TB. The first electronic extraction resulted in a total number of 193 papers. In PubMed after extraction hits, using Boolean extractions and screening it yielded a total of 119 articles, and the same was applied to the Web of Science database which consequently also yielded a total result of 74 papers. We screened both databases using abstracts and titles and handpicked 103 articles after duplication. Sixteen articles were further excluded after screening by abstract and titles. Eighty seven articles were eligible. Furthermore, 54 articles were pulled out after accessing their full text. A second reviewer provided recommendations after going through the list and screening. Twenty eight articles were excluded due to non-relevant outcomes and some study designs were mathematical in a model, which didn't fit our criteria, and some were also excluded due to information overlap. We then thoroughly read the full text and were able to get 26 available articles that met our inclusion criteria during our search (Figure 1). After cross-checking carefully, duplication methods were applied and handpicked to prevent a repetition of a particular study. Longitudinal studies were checked using the PICO element, cross-sectional and observational studies was cross-checked using the STROBE checklist and qualitative papers were crossed checked by the critical appraisal checklist. All the articles screened passed for eligibility. Seven qualitative searches were found, one mixed method of qualitative and quantitative, nine cross-sectional articles, and three cohort studies. We also encountered one case report, one follow-up study, three longitudinal studies, and finally one randomized study. Our study also comprised 15 different countries with three different multi-country studies. Articles concluded that decrement in mental health impacts TBs outcome and compliance hence there is an increase tendency to medication adherence and poor prognosis. With prior inclusion and exclusion criteria, our search was narrowed down to a total of 103 papers.

### Location of Studies

**Figure 2:**
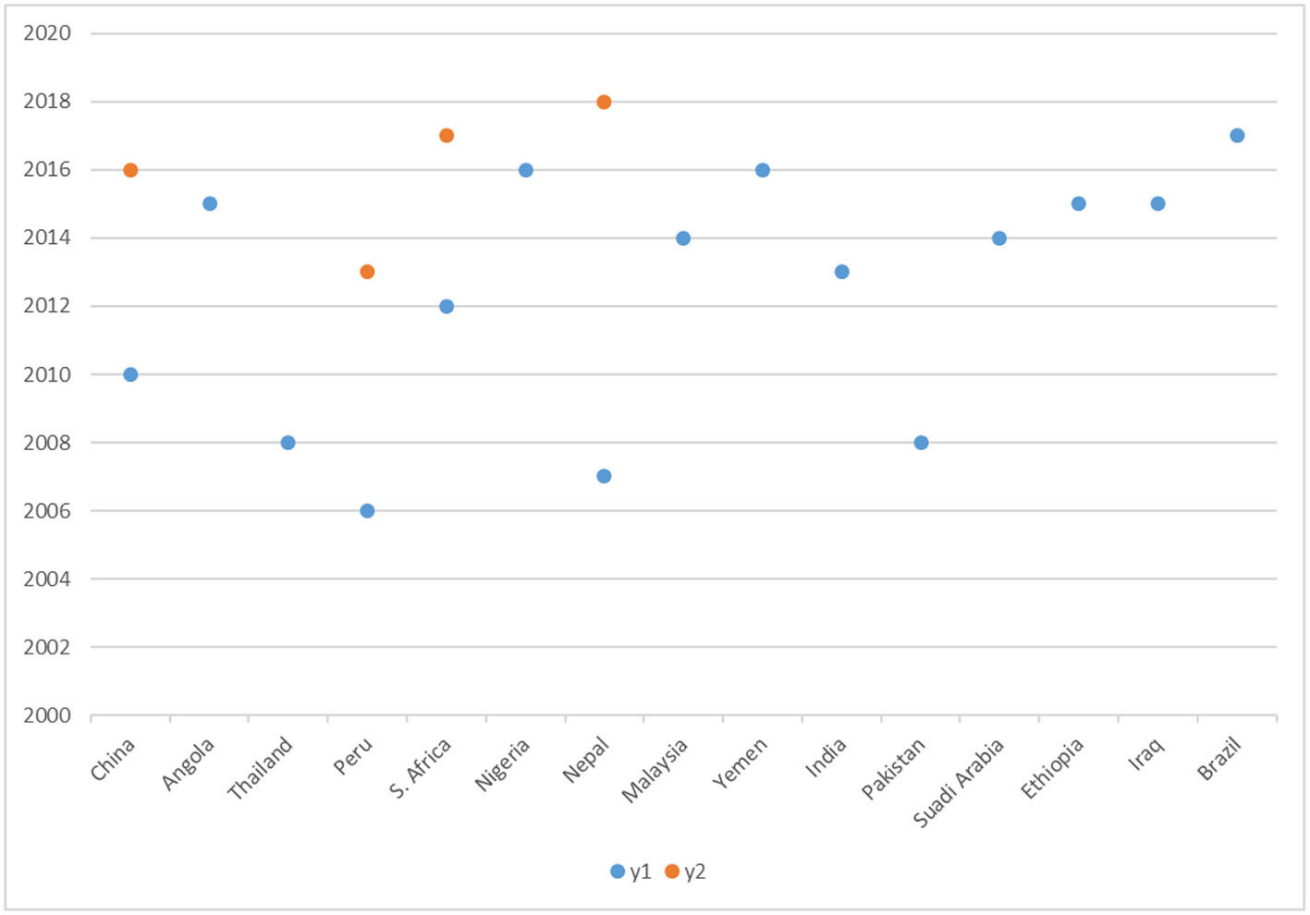
Publications in different regions and in different periods.

Of the 26 studies we analyzed, three were multi-country related, three were in South Africa, two were in Peru, two were in Ethiopia, two were in Nepal, two were in China and the other nine studies were conducted across the globe. Based on the World Bank Report (25), we classified the countries into upper-middle-income countries (UMICs), low-middle-income countries (LMICs), low-income countries (LICs), and high-income countries (HIC). Figure 2 illustrates the publications in different regions and different years.

### Interventional Outcomes

We divided interventional outcomes into depression, anxiety, and social support, and stigma. There were 18 articles reporting depression but only four articles (#1, 3, 21, and 26) adopted interventions, and the outcome of mental health with medication adherence were improved in patients making up 22.2% of total articles in the depression category. The remaining 14 articles (#2, 7, 8, 9, 11, 13, 14, 15, 16, 17, 19, 20, 23, and 25) did not perform interventions but reported data on depression. There were four articles (#1, 3, 21, and 26) adopted interventions and the other 14 articles (#2, 7,8,9,11,13,14,15,16, 17, 19, 20, 23, 25) reported data on anxiety. Regarding social support and stigma, there were 4 articles (5, 10, 12, and 22) that adopted interventions and six articles (#4, 6, 18, and 24) that had no intervention but reported data on social support and stigma. Table 3 gives the summary of the interventions and non-interventional outcomes.

**Table 3:** Interventional outcomes.

**Category of mental health**	**Article number; n%**	**Intervention** **(if applicable)**	**Outcomes (good or bad)**
Depression	1, 3, 21, 26; *n* = 22.22%	Intervene	Improve both physical and mental wellbeing
	2, 7, 8, 9, 11, 13, 14, 15, 16, 17, 19, 20, 23, 25; *n* = 77.77%	No intervention	Compromise mental wellbeing
Anxiety	1, 3, 21, 26; *n* = 22.22%	Intervene	Improve both physical and mental wellbeing
	2, 7, 8, 9, 11, 13, 14, 15, 16, 17, 19, 20, 23, 25; *n* = 77.77%	No intervention	Compromise mental wellbeing
Social support and Stigma	5,10,12,22; *n* = 50%	Intervene	Improve both physical and mental wellbeing
	4, 6, 18, 24; *n* = 50%	No Intervention	Compromise mental wellbeing

### Depression

Depression in TB patients leads to the outcome of delay in seeking medical care which eventually leads to negative medication adherence (6). Psychiatric comorbidity being reported as depression was a major decrement to health leading to a default in seeking appropriate health (26). In a research done in South Africa, a significant frequency of psychological distress among tuberculosis patients was found, with most individuals, particularly those from lower socioeconomic classes, becoming depressed throughout treatment, resulting in a lack of negative health-seeking behavior. (27). Furthermore, patients classified as MDR-TB experienced more depression (28). The impact of mental health on TB patients has hindered their wellbeing both emotionally and spiritually which makes it a hindrance to seeking a healthy behavior, hence affecting treatment outcomes in a long round (29, 30). In Peru, a study reported 37% depression in newly identified TB cases (31). A cross-sectional survey from 48 low and middle-income countries concluded that the proportion of depression is relatively high among TB patients with a consequent decrement in health status than in that among the general population (32). Depression peaks in patients on anti TB medications, hence we need strategic outlines and interventions to deal with the side effects of these medications. But fewer studies have been conducted on this subject matter (29).

### Anxiety

The impact of anxiety has been demonstrated largely through the feeling of anger and ruminating over past events that led to contracting TB which could prolong treatment duration (31). Psychiatric co-morbidity increases the risk to decrement in health and default (26). Anxiety was found to be a key psychological domain in a Zambian study in which 37.6% of patients were found to be suffering from anxiety (31). A similar study conducted in Huambo Angola had similar findings outlining emotional distress and anxiety to be present among TB patients (33). Anxiety disorder and miliary TB were also reported in a case study of a 67-year-old Saudi man who qualified for anxiety definition under the Diagnostic and Statistical Manual of Mental disorders (DSM-5) with excessive anxiety, but his symptoms elevated due to treatment of TB (34). This report limits the studies only to identifying anxiety symptoms in association with TB but couldn't go on further to outline the mechanism involved in it (34). In Porto Algero, Brazil a study also demonstrated generalized anxiety disorder prevalent in TB patients and quantified the number of psychiatry co-morbidity in 68% of hospitalized TB patients (6). Anxiety and mental health disorder have been demonstrated to be in poor accordance with effective treatment outcomes (6). Poor medical compliance and adherence to treatment were also demonstrative in a study conducted in Pakistan with an anxiety rating of 47% (35). Hence anxiety is also a key mental health problem in TB patients.

### Social Support and Stigma

Despite the fear of being infected, family support and individual relationships have proven to help most TB patients overcome the fear of public rejection (36). A study conducted in Peru outlined that all patients with TB who disclosed their disease to their families were welcomed and cherished with an abundance of love, encouragement, financial support, and emotional strength as compared to those who hid it away from their loved ones (17). However, a crucial factor leading to high default of TB care is stigma (37). Stigma negatively affects the individuals by breeding depression, self-hate and disappointment, hindering patients from accessing the required healthcare he/she needs (38). With a good support system, family members and loved ones served as advocates who constantly reminded TB clients to seek help (17). External stigma as well as familial stigma both have huge impacts on the mental health wellbeing of people affected by TB. In the family settings, patients suffering from TB were sometimes left alone to fend for themselves, leading to isolation and self-neglect (37). Stigma and discrimination have led to a negative effect on health-seeking behavior in patients who suspects having TB due to long cough duration (39). Being highly portrayed in communities, especially in low and middle-income countries (LMICs), people suffering from TB tend to hide their symptoms and rather spread the infection (40). A research conducted in Peru assessed treatment outcomes when nurses served as emotional supporters to patients with TB and concluded that social support and networking was vital to the recovery as it helped their spiritual wellbeing (41). It is stipulated by other studies that, socio-cultural attributes of most communities due to lack of public education on TB interfere with treatment adherence and may lead to new infections in the future (42, 43). Social support is hence an important determinant for a good outcome in all diseases. A study from Shaanxi province in China showed how lack of TB education among family members affected the outcome of TB and hence an integral program and campaign was needed to improve the outlook on TB (44, 45). The sparse evidence presented is also an indication of mental health neglect.

### Interventional Approaches

Quality of Life is a multifaceted notion that spans the physical, social, psychological, economic, spiritual, and other realms. As a result, it's difficult to define and measure, but it may be roughly defined as people's judgments of their place in life in relation to their objectives, aspirations, standards, and worries in the context of the culture and value systems in which they live. As part of mitigations to improve patients' quality of life, some countries implemented interventions to combat clinical depression and anxiety observed among TB patients. One study conducted in Malaysia reported addressing mental wellness on time manner by first accessing it at baseline and during the treatment course. This aided early identification of depression and anxiety onset in TB patients for which psychosocial counseling was offered to improve the mental wellbeing (46). Additionally, two studies found that targeted detection and aggressive treatment of major depressive episodes from baseline would also improve the mental wellbeing of TB throughout treatment (31, 34). In Peru, there was a marked increase in mental health after providing necessary support to patients (41). Counselors were also provided in one setting in Nepal, which saw to counseling TB patients to cope with their disease and treatment side effects (47). An encouragement for a high level of support from family and friends also proved important and effective in the recovery process in India (48). Implementing interventions that measure and mitigate stigma at the local community level also proved an effective way of the intervening potential for depression among TB clients as was observed in Thailand (49). These interventions have a good impact on patients' treatment outcomes and quality of life. Hence, we entreat that such interventions will have in-depth research to help identify suitable measures in treating TB patients worldwide.

The requirement for a multidisciplinary team, such as incorporating the clinical psychologist when sent home or during an inpatient, was highlighted in certain literatures. The core of these disciplines will aid in bridging the gap between depression and home-based long-term care. Another intervention that may be used to reach remote settlers is the use of telemedicine. In its most basic form, telemedicine may be used. In the absence of a smart phone, satellite tiny phones can be used to call clients on a regular basis for a phone checkup. This is an interventional strategy that will be quite beneficial. It will also assist if DOT centers are established in health facilities or primary health care institutions. Health care workers who will staff these clinics should be given extra training in basic psychological assessments to aid in the rapid identification of symptoms for appropriate treatment or referral to a specialist (50). The examples above are only a handful of the interventional strategies that are required.

## Discussion

This review ascertained the effects of mental wellbeing on the impact of effective TB treatment around the world. Out of 26 articles retrieved, all articles well documented the observed impacts of mental health on treatment outcomes of TB and consequent decrement in the mental aspects of TB victims. However, limited research existed on the effects of possible interventions to improve the balance between mental wellbeing and improvement of TB outcome (6, 40, 47, 51, 52).

One qualitative study from Peru demonstrated how nurses helped hospitalized MDR-TB patients serving as treatment supporters to cope well with their infirmity and there was significant progress in the outcome of treatment (41). We subsequently think this type of intervention should be applied in different regions across the world to help promote the mental health of TB patients in addition to encouraging family members and immediate friends circle to join in the support. One of the interventions was timely actions taken within the treatment period to address mental wellbeing using feasible tools and designing indicators to help alleviate symptoms (46, 49). Another intervention was to extend educational programs to families and caregivers to encourage patient adherence to seeking positive health behavior (48). Educational programs can take place on different platforms such as social media, radio, and TV programs, marketplace campaigns in LMICs, and involving local celebrities to serve as ambassadors for educating the masses on such issues. We think this could help patients establish strong self-worth. A follow-up study in Penang General Hospital using SF-36 questionnaire found that even though other symptoms improved significantly with treatment, 23% of patients were at risk of depression at the end of TB treatment, which may be due to lack of psychological support during the treatment period (46). This implies that assessing psychological status at baseline and working toward improving every domain of HRQOL is significant in the pathway to a free TB world in the near future. We suggest that hospitals should include psychological therapy such as counseling as part of the treatment program. In another study, major depressive and anxiety episodes (MDE) were measured using a 5-item version in 325 patients (31). The presence of major depressive episodes at baseline was associated with negative outcomes (31). Depressive and anxiety are mostly found during an initial stages of diagnosis and may lead to negative outcomes if psychological packages are not strategized to encourage patients to adhere and retain care regimes (14, 48).

HRQOL in TB patients is usually underestimated in many settings, especially in LMICs where major resources are usually directed to improving only microbial symptoms, but neglecting the mental, physical and other domains in relation to HRQOL. A South Africa study adopted the SF-12, EQ-5D and St. George's respiratory scale, and observed a positive relationship between treatment adherence and HRQOL in patients with TB (53). It is therefore obvious that elevating microbial symptoms alone by using drugs will address some domains but others may be left out. LMICs with a high burden of TB may prioritize anti-TB drugs over psychological support. We think this phenomenon may be due to an extra financial burden that will be invested into getting more mental health workers which might not fit into the budget plan of LMICs. Also, a prospective cohort study in Nepal suggested psychological support package is acceptable to patients and hence requires additional investment to counselors in TB clinics (47). This supports the recommendation that there is an urgent need for resource allocation to psychological support training in clinics and DOT centers in LMICs.

There is a need for further studies and improvement on caregivers training in recognizing, identifying, and implementing a social network approach to treating TB patients. National and provincial/district CDCs, primary care facilities, and other public health agencies have to mobilize workforces that will look into all aspects of health during the treatment course. Extra funding and research are needed, especially in the field of mental health. An integral effort by all agencies to increase the capacity of mental health workers in the field and national TB programs and the need to support primary caregivers should also be frontier on the agenda in most countries. Catastrophic cost on the victim's finances and family, socio-economic status considerations in that area of study, and cultural beliefs of the society in question are some of the driving factors we identified that could also lead to a decrement in the mental wellbeing of patients.

We further assessed the quality of reviewed studies and observed some limitations with some articles which may have affected the quality of the findings. For starters, interpreted findings in some articles were not expressed clearly due to data not being solely collected for analyzing the effect of mental wellbeing on medication adherence. Also, some studies used different measurements for assessing the mental wellbeing of patients. Some articles investigated just specific effects of how TB affects them mentally, while others considered measuring other factors leading to a potential decrease of mental wellbeing among TB patients. Other studies also focused on the measurement of physical wellbeing as compared to mental wellbeing but mostly neglected to make it an exclusive inclusion of all factors leading to medication adherence. This review also observed that qualitative studies mostly collected data only at one point in time without follow-up. We deem this an important gap as results may not reflect the true context of mood alterations, which could have led to a bias.

## Limitation and Strength

This systemic review was limited in a number of ways. The search was limited to only 13 years from 2005 to 2018. During the search, we might have limited ourselves to only papers which were available in the databases used and their titles as well as abstracts matched our keywords. Moreover, taking data samples representing the whole population is very vital to understanding the variation and complexity of studies. The use of convenient sampling however seemed to be commonly utilized in most research and is a vital limitation to most studies (6, 17, 54). Representation of just a particular group of people, for example, smear-positive TB patients alone, cannot be used as a generalization for the whole population (46). Studies that were cross-sectional in nature did not really identify a clear-cut relation between anxiety, depression with reference to other factors that have been found to compromise the mental wellbeing (53). They were mostly focused on the determination of a link between depression, anxiety and TB excluding confounding factors that may contribute to a compromised mental health (53). Considering general factors will help identify areas to be addressed so as to get a quick intervention in that area. The correlation of anxiety, depression, and TB has been established in most studies but factors have been poorly outlined (52).

Irrespective of the above-noted limitations to the quality of the articles, a review of the data collated by them is highly informative and good enough to inform treatment policy formation and upgrade. This review combined findings from a case report, qualitative and quantitative studies together thereby ensuring the capture of all relevant information on the topic making it one of the strengths.

## Conclusion

The evidence of the effects of mental health on TB outcomes has been well captured in this review. Depression, anxiety and poor social support, and stigma evidently affect the mental well-being of individuals across the globe, irrespective of age, race, demographic characteristics, geographical location, and social status. Moreover, there is an urgent need to address TB in a multidisciplinary approach to combat the overall quality of treatment outcomes. Patients with all forms of TB may or may not experience optimum mental wellbeing depending on a variety of reasons influencing the patient and this needs further research.

### Implication of Research

This review underscores the importance of studies in the field of mental health in relation to TB patients across the globe. Further research needs to be conducted in the field of mental health to outline strategies that will help promote the mental wellbeing of TB patients during treatment across the world. Furthermore, recent research on mental health and its association to medication adherence using causal inference analysis and meditational modeling will optimize treatment protocols for better outcomes as well as help understand limitations and pathways leading to default in TB treatment.

## Data Availability Statement

The original contributions presented in the study are included in the article/Supplementary Material, further inquiries can be directed to the corresponding author.

## Author Contributions

CA and JW: conceptualization, methodology, and software. CA: formal analysis and visualization. CA, MM, BH, QL, and HS: investigation and writing—original draft preparation. MM, BH, QL, and HS: data curation and validation. JW: supervision, project administration, resources, writing—review and editing, and funding acquisition. All authors have given final approval of the version to be published and agreed on the journal to which the article has been submitted.

## Funding

This study was funded by the National Natural Science Foundation of China (81973103), Nanjing Major Science and Technology Project (2021-11005), Medical Research Project of Jiangsu Health Commission (ZDB2020013), and Key Project of Philosophy and Social Science Research in Colleges and Universities in Jiangsu Province (2020SJZDA096). The funding agencies had no role in the study design, data collection, analysis, decision to publish, or preparation of the manuscript.

## Conflict of Interest

The authors declare that the research was conducted in the absence of any commercial or financial relationships that could be construed as a potential conflict of interest.

## Publisher's Note

All claims expressed in this article are solely those of the authors and do not necessarily represent those of their affiliated organizations, or those of the publisher, the editors and the reviewers. Any product that may be evaluated in this article, or claim that may be made by its manufacturer, is not guaranteed or endorsed by the publisher.
