# Supplementary material for: Mental Health Status and Its Impact on TB Treatment and Its Outcomes: A Scoping Literature Review
Source: Front Public Health. 2022 May 31;10:855515. doi: 10.3389/fpubh.2022.855515 (PMC9194388; doi:10.3389/fpubh.2022.855515)
Supplement: Supplementary file 1 [file Table_1.docx]

**Supplementary file 1.** The search strategy for identification of relevant studies

**Web of science.**

**Search Used.**

1. (Topic= “Tuberculosis”) OR (Topic= “TB”)
2. (Topic= “Mental Health”) OR (Topic= “Mental status”)
3. (Topic= “Depression”)
4. (Topic= “Anxiety”)
5. (Topic= “Social Support and Stigma”)
6. (Topic= “Treatment Outcome”)
7. Results hit= (943)

**PubMed**

**Search Used**

(“Tuberculosis” [Mesh] OR TB [Mesh] “Mental Health” [Mesh])

Advanced search.

1. Click on advance search. (all fields)
2. Type in Mental health and click on search and add to history.
3. Type in Depression and click on search and add to history.
4. Type in Anxiety and click on search and add to history.
5. Type in Social support and stigma and click on search and add to history.
6. Type treatment outcomes and click search and add to history.
7. After all results pop out, use Boolean operators AND to combine all search topics
8. Mental health And Depression AND Anxiety AND Social Support and Stigma AND Treatment outcomes.
9. Results hit= (1029)
