# Supplementary material for: Mental Health Status and Its Impact on TB Treatment and Its Outcomes: A Scoping Literature Review
Source: Front Public Health. 2022 May 31;10:855515. doi: 10.3389/fpubh.2022.855515 (PMC9194388; doi:10.3389/fpubh.2022.855515)
Supplement: Supplementary file 2 [file Table_2.docx]

**Supplementary file 2.** Quality Assessment checklist for qualitative, cross sectional, longitudinal and reviews

**Type of studies Number of studies Article number Checklist Ratio score**

(0 - 5)

1. Qualitative studies 7 1,6,7,33,15,22,32 Glasgow Critical 4.0

Appraisal

1. Cross sectional 11 9,11,14,18,19,20,21, STROBE 4.5

23,29,31,30

1. Cohort Studies 3 4,10,16 - -
2. Mixed Qualitative & 2 12,25 - -

Quantitative Studies

1. Literature Review 2 13,14 PRISMA 4.5
2. Systemic Review 2 2,27 PRISMA 4.8
3. Case Report 1 3 - -
4. Follow Up 1 5 - -
5. Longitudinal Studies 3 17,24,28 PICO 4.5
6. Randomized 1 1 - -
