# Supplementary material for: Mental Health Status and Its Impact on TB Treatment and Its Outcomes: A Scoping Literature Review
Source: Front Public Health. 2022 May 31;10:855515. doi: 10.3389/fpubh.2022.855515 (PMC9194388; doi:10.3389/fpubh.2022.855515)
Supplement: Supplementary file 3 [file Table_3.docx]

**Supplementary file 3.** Studies involved in this review

**Category of Number of Articles Intervention Outcomes**

**Mental Health n% (If applicable) (Good or bad)**

1. Depression 2, 3,5,25,28 Intervene Improve both physical and

n=21.7% Mental well-being

4, 9,10,11,14,16,17,18 No Intervention Compromise Mental well-being

19,20,21,23,24,26,27,30,31,33

n=78.26%

1. Anxiety 2, 3,5,25,28 Intervene Improve both physical and

n=21.7% Mental well-being

4, 9,10,11,14,16,17,18 No Intervention Compromise Mental well-being

19,20,21,23,24,26,27,30,31,33

n=78.26%

1. Social Support 7,12,15 29 Intervene Improve both physical and

And Stigma n=40% Mental well-being

6, 8,13,22,27, 32 No Intervention Compromise Mental well-being

N=60%
